# Papules du dos des mains: penser au granulome annulaire

**DOI:** 10.11604/pamj.2017.27.26.7543

**Published:** 2017-05-10

**Authors:** Hasnaa Zaouri, Baderddine Hassam

**Affiliations:** 1 Service de Dermatologie-vénéréologie, Centre Hospitalier Universitaire Avicenne, Rabat, Maroc

**Keywords:** Papules des mains, granulome annulaire, dermocorticoïde, Granuloma annulare, papules on hands, dermocorticoids

## Image en médecine

Le granulome annulaire est une dermatose inflammatoire chronique. Les théories physiopathologiques actuelles incluent une réaction inflammatoire de type Th1 et une altération du tissu élastique. Cliniquement, ce sont des papules asymptomatiques, de couleur chaire ou rosée, avec une bordure papuleuse annulaire. IL siège en regard des saillies articulaires des mains ou pieds. Sur le plan histologique, on note la présence d'une nécrobiose centrale, entourée d'un infiltrat histiocytaire palissadique. L'étiologie de cette dermatose est inconnue. Certains facteurs ont été incriminés tels que les piqûres d'insectes, les traumatismes, les infections virales, ainsi que la prise médicamenteuse. L'association aux néoplasies et au diabète a été rapportée. Le traitement repose sur la cryothérapie ou la corticothérapie locale pour les formes localisées; la corticothérapie systémique, les antipaludéens de synthèse sont discutés pour les formes disséminées. Nous rapportons le cas d'une patiente de 29 ans, qui consulte pour des papules asymptomatiques du dos des mains évoluant depuis 3 mois. L'examen clinique trouve des papules arrondies de couleur chaire, de 4 mm de diamètre au niveau de la face dorsale des mains (A&B). Les diagnostics évoqués sont le lichen plan, les nodules de Darier Roussy, l'érythéma élévatumdiutinum et le granulome annulaire. L'examen histologique a objectivé une dermite granulomateuse de disposition interstitielle en faveur d'un granulome annulaire (C). Le bilan étiologique demandé est normal. La patiente a été mise sous dermocorticoïde classe forte à raison d'une fois par jour. L'évolution était marquée par la disparition complète des lésions après 4 semaines (D).

**Figure 1:**
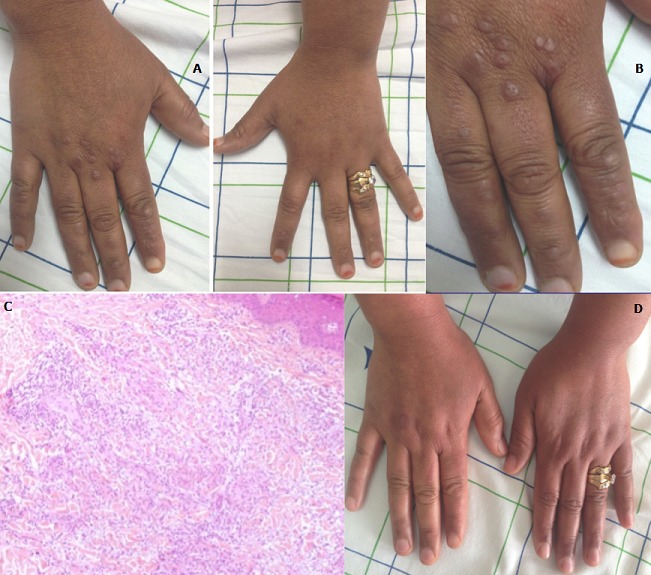
(A) papules au niveau de la face dorsale des mains; (B) papules arrondies de couleur chair, de 4 mm de diamètre au niveau des mains; (C) coloration HES; G×50: dermite granulomateuse de disposition interstitielle; (D) disparition complète des lésions après traitement

